# Information preferences about treatment options in diffuse cutaneous systemic sclerosis: A Delphi consensus study

**DOI:** 10.1177/23971983211043311

**Published:** 2021-09-08

**Authors:** Julia Spierings, Hilde Nienhuis, Eva van Lieshout, Jacob M van Laar, Arwen H Pieterse

**Affiliations:** 1 Department of Rheumatology and Clinical Immunology, University Medical Centre Utrecht, Utrecht, The Netherlands; 2 Centre for Rheumatology and Connective Tissue Diseases, Department of Inflammation, Division of Medicine and Royal Free, UCL Medical School, University College London, London, UK; 3 Department of Internal Medicine, University Medical Centre Utrecht, Utrecht, The Netherlands; 4 Department of Biomedical Data Sciences, Leiden University Medical Centre, Leiden, The Netherlands

**Keywords:** Systemic sclerosis, scleroderma, Delphi method, information provision, patient education, health communication

## Abstract

**Objectives:**

The aim of this study was to identify and prioritize aspects essential for decision making in patients with diffuse cutaneous systemic sclerosis (dcSSc) and to gain insight into information preferences of treatment options which could guide development of a leaflet for patients.

**Methods:**

A three-round Delphi study was conducted with a panel of patients with dcSSc. The questionnaire was based on a systematic literature search regarding benefits and harms of four main treatment options in dcSSc: methotrexate, mycophenolate mofetil, cyclophosphamide pulses and stem cell transplantation. Patients were asked to identify information that is essential for making a treatment decision. After the third round, a live, online discussion was held in order to reach consensus on these items and to discuss the content and design of the leaflet. Consensus was defined as ⩾75% agreement among panel members.

**Results:**

Of the 36 patients invited, 78% (n = 28) participated in one or more rounds, 67% (n = 24) completed the first, 69% (n = 25) the second and 75% (n = 27) the third round. In the last round, median age of participants was 51 years (interquartile range, 18) and median disease duration 4 years (interquartile range, 5); 52% were female. Patients had been treated with mycophenolate mofetil (67%), methotrexate (44%), cyclophosphamide (41%), autologous stem cell transplantation (26%), rituximab (4%) or were treatment-naïve (7%). Eight patients joined the live panel discussion. The panel reached consensus on seven benefits (prolonged progression-free survival, improved quality of life, improved daily functioning, improved pulmonary function, improved skin thickness, improved mobility and reduced fatigue) and four harms (treatment-related mortality, infections, cardiac damage, increased risk of cancer) as essential information for decision making. Also a design of a leaflet was made.

**Conclusion:**

This study identified information about treatment options in dcSSc that should be addressed with patients. Our results can be used to develop effective patient information.

## Introduction

Diffuse cutaneous systemic sclerosis (dcSSc) has a high impact on quality of life and is associated with increased mortality.^1^ Several treatment options are available in dcSSc, including immunosuppressive medication (such as antimetabolite and alkylating agents), and hematopoietic stem cell transplantation (SCT).^2–5^ The choice of treatment depends on preferences of both the rheumatologist and the patient. Among rheumatologists, there is a fair level of agreement regarding treatment strategies for some aspects of SSc.^6^ Still, patient preferences should be taken into consideration when choosing a treatment and shared decision making (SDM) is key in choosing the appropriate treatment strategy.

A study in patients with SSc and interstitial lung disease showed, however, that in most consultations, patients were given little opportunity to explain their concerns about the disease and possible treatments.^7^ Furthermore, in a qualitative study investigating the decision-making process in dcSSc, patients reported that the lack of accessible, reliable, dcSSc-specific patient education about treatment options is currently complicating decision making.^8^ In order to optimize the SDM process in dcSSc, patient information that provides a clear overview of treatment options needs to be developed.^9^ To create patient information that meets the needs of patients, more insight is needed in information preferences of patients with dcSSc.^10,11^ The aim of this study was to identify and prioritize aspects that should be included in patient information on treatment options in dcSSc.

## Methods

### Design

This is a consensus study using Delphi technique. In this structured process, we used three online questionnaire rounds and one live, online panel meeting with a final voting round in order to reach consensus.^12–14^ This study was classified by the institutional review board as exempt from the Medical Research Involving Human Subjects Act (19-666/C). Written informed consent was obtained from all participants.

### Participants

All patients with an established diagnosis of dcSSc treated at the University Medical Centre Utrecht, The Netherlands, between 1 January 2010 and 1 February 2020, were selected from the electronic hospital records. These patients were invited by their rheumatologist to participate in the study. All selected patients were verbally informed about the study by their rheumatologists (J.S., J.M.v.L.) during a routine hospital visit or by phone. When interested, they received written patient information by email and a link to the first Delphi round. Informed consent was obtained by an online form. We estimated that at least 25 participants were needed for a representative panel.^15^ Non-responders received a reminder 10 days after the invitation was sent.

### Questionnaire design and content

The questionnaire was based on a systematic literature search on benefits and harms or side-effects of the four main therapies used in dcSSc, namely methotrexate, mycophenolate mofetil (MMF), cyclophosphamide (CYC) and autologous SCT.^13,14^ Results of this literature search are summarized in Supplementary Appendix 1.

Round 1 consisted of a list of benefits and harms of the four treatment options. All questions and information about items was provided in simple text in order to make it easy to read for lay persons.

Participants were asked to assess whether the given benefit or harm should be discussed during decision-making consultation (essential or not necessary). For each harm and benefit, the prevalence belonging to each of the treatment options was given, to the extent reported in the literature. Participants could also suggest new information items. The questionnaire was pilot-tested by two patients prior to the first round.

In the second and third rounds, suggestions from participants from the prior round were added to the list of items. In the third round, only the items with no consensus were shown. Also the percentage of participants considering the item as essential was reported. Participants were now asked to choose the five most important benefits and five most important harms that should be discussed during an informed consent consultation. The results from the second round were shown as a percentage in the third round, in order to let participants reconsider their previous response.

Socio-demographic (age, sex and education level) and disease-related details (disease duration and treatment history) were collected with multiple-choice questions the first time a patient participated.

### Consensus meeting

After the third online round, a live, online consensus meeting using the videoconferencing platform Zoom© was held to discuss the items for which consensus had not yet been reached. This was done online to enable patients with travel limitations to join the discussion and to limit health risks during the COVID-19 pandemic. In rounds 2 and 3, and in the live session, consensus was considered to be achieved when ⩾75% of the panel ticked the same answer. In this meeting, content of an information leaflet about treatment options for patients was discussed as well.

### Data collection and analysis

In rounds 1–3, data were collected using the software programme Calibrum®. Descriptive statistics were used to report patients’ characteristics. Fisher’s exact tests were used to compare categorical variables sex and treatment history with items quality of life, fatigue, skin thickness, daily functioning, infertility, hair loss and risk of malignancies and impact on social activities. The Mann–Whitney U test was used to assess the relation between disease duration and these prioritized items. A two-sided p-value of ⩽0.05 was considered statistically significant. Statistical analysis was done using SPSS version 25.0.

## Results

### Participants

For all three rounds, 36 patients were invited to participate. In round 1, 24 patients completed the survey (67%); in round 2, 25 patients responded (69%); and in the last round, 27 patients responded (75%). Of the 27 patients who participated in the third round, 14 (52%) were female, median age was 51 years and median disease duration was 4 years. MMF was included in the treatment history of 67%, methotrexate in 44%, CYC in 41% and autologous SCT in 26% of all participants. For an overview of all patient characteristics, see Table 1. Eight patients participated in the live online panel discussion.

**Table 1.** Patient characteristics.

	N = 27 (%)
Age in years (mean, SD)	53 (11)
Female sex	14 (52)
Highest completed educational level	
• Low (no/primary school)	–
• Intermediate (secondary school/vocational training)	16 (70)
• High (high vocational training/University)	8 (30)
Disease duration in years (median, IQR)	4 (5)
Treatment history	
• MMF	15 (56)
• MTX	12 (44)
• CYC	10 (37)
• SCT	9 (33)
• Rituximab	1 (4)
• Other	1 (4)
• None	3 (11)

SD: standard deviation; IQR: interquartile range; MMF: mycophenolate mofetil; MTX: methotrexate; CYC: cyclophosphamide; SCT: stem cell transplantation.

### Prioritized information

In the first round, no benefits or harms were dismissed from the list. Five benefits (improved cardiac function, oesophageal function, improved mobility, improved mood, improved fatigue) and six harms (erectile dysfunction, neuropathic pain (CYC), temporary negative impact on social life (SCT), mobility (SCT), hormonal imbalance (SCT), increased fatigue (SCT and CYC)) were suggested by patients in the free-text fields. These items were added to the second round. In the second and third rounds, the items were prioritized. After the third round, agreement was reached over the following items: progression-free survival, quality of life, daily functioning and treatment-related mortality (Supplementary Appendix 2). After the live online discussion with the panel, a final list was made with the benefits and harms that were considered most important to share with patients (Table 2). Patients reached consensus on seven benefits and four harms as essential information for the decision-making process. The benefits included prolonged progression-free survival, improved quality of life, improved daily functioning, improved pulmonary function, improved skin thickness, improved mobility and reduced fatigue. The four harms included treatment-related mortality, infections, cardiac damage and increased risk of cancer.

**Table 2.** Agreement on benefits and harms that should be addressed.

Benefits	Agreement	Harms/cons	Agreement
**Improved pulmonary function**	**100%**	**Infections**	**88%**
**Improved skin thickness**	**100%**	**Cardiac damage**	**88%**
**Improved mobility**	**100%**	**Higher risk to develop malignancy**	**88%**
**Progression-free survival**	**95%** ^a^	**Treatment-related mortality**	**79%** ^a^
**Improvement of quality of life**	**77%** ^a^	Flare or relapse of disease	63%
**Improved daily functioning**	**77%** ^a^	Temporary increase in fatigue	25%
**Improved fatigue**	**75%**	Temporary negative impact on social activities	25%
Improved hand function	25%	Nausea	25%
Improved mood	25%	Other autoimmune disease	13%
Improved cardiac function	13%	Hormonal imbalance	13%
Improved oesophageal function	7%	Less ability to focus	13%
		Diarrhoea	13%
		Erectile dysfunction	13%
		Depression	13%
		Infertility	13%
		Hair loss	13%
		Haemorrhagic cystitis	0%
		Increased disease activity (granulocyte colony stimulating factor)	0%
		Neuropathic pain	0%
		Cytokine storm	0%
		Mouth ulcers	0%
		Temporary decreased mobility	0%

a Items on which consensus was reached in round 3.

*In bold the items are the panel reached consensus on (* ⩾*75%)*﻿.

Except for erectile dysfunction, men did not prioritize different items compared to female participants. Patients prioritizing infertility had a shorter disease duration (3 vs 15 years, p = 0.015). No significant differences were observed between information preferences and treatment history.

### Information leaflet about treatment options

**Figure 1.**
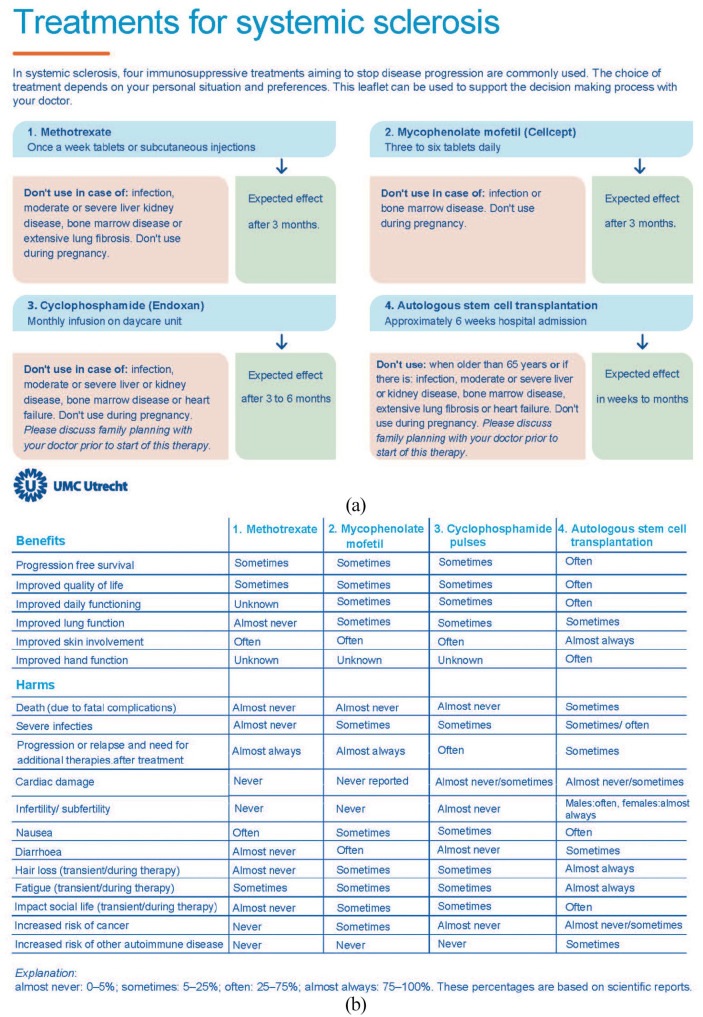
(a) Information leaflet about treatment options (part 1) and (b) information leaflet about treatment options (part II).

In addition to the items marked as essential for making the treatment decision, the panel in the live online session (N = 8) agreed that all items prioritized by at least one patient in the online rounds should preferably be included in the information leaflet. They also suggested to include information about different routes of administration, the time to a noticeable treatment effect and contraindications for the four treatments. The final version of the leaflet was developed and send to the panel for feedback and approval (Figure 1).

## Discussion

In this study, we identified and prioritized information about treatments that is central for patients with dcSSc in decision making. Patients reached consensus on seven benefits and four harms as essential for decision making about treatment.

Most of the benefits that were prioritized are related to quality of life and daily functioning (progression-free survival, improved quality of life, daily functioning and mobility and reduced fatigue). These findings are in line with patient perceptions about essential outcomes of treatment in SSc and other rheumatic diseases, which showed that quality of life and daily functioning were highly prioritized.^16,17^

There were three rounds and a live online meeting needed to reach agreement on the items, which reflects the different views of individual patients on this subject. Furthermore, no agreement was reached on items about infertility and disease relapse, which are relatively common adverse events for CYC and SCT with considerable impact on quality of life. Patients prioritizing infertility had a significant shorter disease duration. Insight in information needs of subgroups might be helpful to provide tailored information. For example, in oncology, different information preferences were observed in female versus male, and in younger versus older patients.^18^ In patients with diabetes mellitus, different information needs were found in subgroups including age, education level and disease duration.^19^ In our study, no differences in information preferences were observed between patients from different age groups, sex or with different treatment histories. This can be explained by the relative small subgroups and small range in age and requires further research in larger groups.

In addition to optimal information content, we anticipate that effective information provision also includes structuring and tailoring information. In the last decades, patient decision aids have been developed, which provide more individualized information about therapeutic options for various conditions. In a previous study in patients with systemic lupus erythematosus (n = 298), effectiveness of a patient decision aid was compared with an information leaflet. As a result, the choice of treatment was much more aligned between patients and physicians when using the decision aid. Also, the information in the decision aid was rated as being of higher quality compared to usual care.^20,21^ Our study findings and the designed leaflet are generic and are meant as a first and hopefully helpful evidence-based guideline to inform patients when they face a treatment decision. Additionally, we realize that information, especially about risks, can be hard to understand and therefore health care professionals should preferably provide additional explanation.^22–24^

Our study is the first to investigate information preferences in patients with dcSSc, but has some limitations. First, we selected the benefits and harms to be included in the first questionnaire ourselves. To that end, we have conducted an extensive literature search, so we are confident that we included the most relevant and evidence-based information items, but we realize we may have missed some. For that reason, we invited the panel to add items in the first Delphi round. Also, we appreciate that the patient panel might not be representative for all patients with dcSSc because this study was conducted in a tertiary centre and patients who are willing to participate to online questionnaires and an online live discussion might have different information preferences than patients who do not. Third, the education level of our participants was generally high, which may have resulted in a leaflet that may be too difficult to understand for patients with lower levels of literacy, as was seen in a study on the readability of information material developed by rheumatologists in other countries.^25^ Further evaluation of the leaflet and information preferences in patients with a lower education level are therefore needed. As a next step, further research will be done on information preferences in a larger group, including patients from other centres, to investigate differences in preferences in subgroups such as sex, age groups, education level and disease stage. Also, an interactive decision aid will be developed and evaluated.

To conclude, we identified information preferences of patients with dcSSc that need to be addressed for optimal participation in treatment decision making. The results of this study were used to develop an information leaflet that can be used in routine clinical practice. Further research on information preferences in subgroups and development of an interactive patient decision aid are now needed to further improve patient education and SDM.

## Supplemental Material

sj-pdf-1-jso-10.1177_23971983211043311 – Supplemental material for Information preferences about treatment options in diffuse cutaneous systemic sclerosis: A Delphi consensus study Click here for additional data file. Supplemental material, sj-pdf-1-jso-10.1177_23971983211043311 for Information preferences about treatment options in diffuse cutaneous systemic sclerosis: A Delphi consensus study by Julia Spierings, Hilde Nienhuis, Eva van Lieshout, Jacob M van Laar and Arwen H Pieterse in Journal of Scleroderma and Related Disorders
