# Supplementary material for: Information preferences about treatment options in diffuse cutaneous systemic sclerosis: A Delphi consensus study
Source: J Scleroderma Relat Disord. 2021 Sep 8;7(1):42–8. doi: 10.1177/23971983211043311 (PMC8922679; doi:10.1177/23971983211043311)
Supplement: sj-pdf-1-jso-10.1177_23971983211043311 – Supplemental material for Information preferences about treatment options in diffuse cutaneous systemic sclerosis: A Delphi consensus study [file sj-pdf-1-jso-10.1177_23971983211043311.pdf]

Supplementary appendix 1

1.1. Literature search strategy

Database: MEDLINE and Embase Search Strategy:

Treatment effects
1 ('Systemic sclerosis' or 'scleroderma')
2 (‘Autologous stem cell transplantation’ or ‘Stem cell transplantation’) OR (‘Methotrexate’) OR (‘mycophenolate mofetil’ or ‘mycophenolic acid’ or ‘Cellcept’) OR (‘cyclophosphamide’ OR ‘endoxan’) OR ('disease modifying antirheumatic drugs’ OR DMARDs)
3. (‘Efficacy’) OR (‘effects’) OR (‘survival’) OR (‘Quality of Life’ or ‘QoL’) OR (‘skin thickness’ or ‘skin score’ or ‘modified Rodnan Skin Score’ or ‘skin’) OR (‘daily functioning’ or ‘daily activities’ or ‘Scleroderma Health Assessment Questionnaire’) OR (‘lung function’ OR ‘Interstitial lung disease’) OR (‘relapse’ OR ‘disease progression’)
4.1 and 2
5.1 and 3
6. limit to 2000/01/01-2020/07/12 and english papers only

Side effects
1 ('Systemic sclerosis' or 'scleroderma')
2 (‘Autologous stem cell transplantation’ or ‘Stem cell transplantation’) OR (‘Methotrexate’) OR (‘mycophenolate mofetil’ or ‘mycophenolic acid’ or ‘Cellcept’) OR (‘cyclophosphamide’ OR ‘endoxan’) OR ('disease modifying antirheumatic drugs’ OR DMARDs)
3. (‘adverse events’ or ‘side effects’ or ‘complications’ or ‘toxicity’) OR (‘Treatment related mortality’) OR (‘infection’) OR (‘fertility’) OR (‘mucositis’ or ‘nausea’ or ‘diarrhoea’) OR (‘lung function’ OR ‘Interstitial lung disease’)
4.1 and 2
5. 2 and 3
6. limit to 2000/01/01-2020/07/12 and english papers only

1.2. Results of literature search on benefits and harms of treatment options in diffuse cutaneous systemic sclerosis

**Treatment**	**Short name**	**Description**	**Results**	**Sources**
SCT				
**Benefits**	Progression/ Event free survival	Improved survival, overall and without occurrence of severe events (for controlled trials: compared to control group)	EFS (0 vs 90%) at 12m[1]<br>OS 74%,EFS 65% at 10yrs[2]<br>OS (86 vs 51%) at 54m[3]<br>EFS (74 vs 47%) at 54m<br><br>OS RR 0.5 [95% CI 0.33 to 0.75][4]<br>OS 85%,EFS 57% at 5 yrs[5]<br>OS 93%,EFS 40% at 10yrs[6]<br><br>EFS 76% at 10yrs[7]	Burt et al. Lancet. 2011<br>Van Laar et al. JAMA 2014<br>Sullivan et al. NEJM 2018<br><br><br>Shouval et al. Biol. Blood Marrow Transplant 2018<br>Vonk et al. Ann. Rheum. Dis 2008<br>Nakamura et al. Mod. Rheumatol. 2018<br>Bijnen et al. Ann.Rheum.Dis. 2020
	Reduced skin thickness	Improvement of skin thickness and extent of skin involvement assessed using the mRSS	Improved mRSS compared with controls (MD 10.62 [95%CI, –14.21 to –7.03])<br><br>Median decreased from 26 to 6 at 5 yrs<br><br>57% > 50% reduction	Shouval et al. Biol. Blood Marrow Transplant 2018<br><br><br>Bijnen et al. Ann.Rheum.Dis. 2020<br><br>Nakamura et al. Mod Rheumatol. 2018
	Improved QoL	Improvement of QoL after treatment assessed using the SF-36 or EQ-5D	Improvement of QoL, particularly the physical domains in several studies, impact on mental QoL is inconsistent.[8]<br><br>56% improvement physical, 31% mental domains SF-36	Puyade et al. Rheumatology 2020<br><br>Shouval et al. Biol. Blood Marrow Transplant 2018<br><br>Sullivan et al. NEJM 2018

	Reduced disability	Improvement in daily functioning after treatment, assessed using the HAQ-DI or SHAQ.	Improved scores SHAQ<br><br>53% had improved score	Puyade et al. Rheumatology 2020<br><br>Sullivan et al. NEJM 2018
	Improved interstitial lung disease/lung fibrosis	Improvement of lung function, extent on imaging or symptoms.	Improved FVC compared with controls MD 9.58 [95% CI, 3.89-15.18]<br><br>FVC improved in 33%, no change 42%, worse 25%.<br>DLco improved in 11%, no change 53%, worse 36%.<br><br>Median FVC and DLco increased from 84% and 55% to 94% and 61% at 5yrs<br>Median Goh scores improved from 14% to 8% at 5 yrs	Shouval et al. Biol. Blood Marrow Transplant 2018<br><br><br>Sullivan et al. NEJM 2018<br><br><br><br>Bijnen et al. Ann Rheum Dis. 2020
	Improved hand function	Improved hand mobility and function, measured with the mHAMIS or subdomain of the SHAQ.	-	-
**Harms**	Treatment related mortality (short term)	Fatal complications related to the treatment.	10% at 24m<br>6% at 54m<br>7% at 10yrs<br>22% at 15m[9]<br>6% at 5yrs[10]<br><br>11% at 5yrs<br>RR 9.00 [95% CI,1.57 to 51.69]	Van Laar et al. JAMA. 2014<br>Sullivan et al. NEJM 2018<br>Nakamura et al. Mod Rheumatol. 2018<br>Helbig et al, Clin Rheumatol. 2018<br>Del Papa et al. Bone Marrow Transplant. 2017<br>Bijnen et al. Ann Rheum Dis. 2020<br>Shouval et al. Biol. Blood Marrow Transplant 2018
	Cytokine storm (short term)	A severe immune reaction in which the body releases too many cytokines as a response to the treatment. Symptoms include high fever, inflammation and sometimes organ failure.	4% (estimation based on described complications in supplementary files)<br><br>3%	Van Laar et al. JAMA 2014<br><br><br><br>Sullivan et al. NEJM 2018
	Increased skin tightness due to G-CSF (short term)	Skin progression related to the growth factors administered during mobilisation.	-	-
	Infections (short term)	Occurrence of infections after treatment.	CMV: 11%<br>EBV: 8%<br>VZ: 4%<br>HSV: 14%<br><br>Any infection: 62%<br>VZ: in 39%<br>CMV: 12%<br>Influenza: 6%<br>Infections (general): in 33%	Van Laar et al. JAMA 2014<br><br><br><br><br>Sullivan et al. NEJM 2018<br><br><br><br>Burt et al. Lancet 2011
	Hair loss (short term)	Temporarily hair loss after treatment.	Almost all patients	Based on side effects reported for used drugs during SCT in non-scleroderma population
	BK infection (short term)	Reactivation of the BK virus leading to (hemorrhagic cystitis)	6%<br><br>17%[11]<br>6%	Del Papa et al. Bone Marrow Transplant. 2017<br>Farge et al. Br J Haematol. 2002<br>Sullivan et al. NEJM 2018
	Nausea (short term)		Almost all patients	Based on side effects reported for used drugs during SCT in non-scleroderma population
	Mouth ulcers (short term)	Mucositis	17%<br>6%	Farge et al. Br J Haematol. 2002<br>Del Papa et al. Bone Marrow Transplant. 2017
	Cardiac toxicity (short term)	Reduced LVEF due to treatment, cardiovascular grade 3 / 4 toxicity	6%<br><br>17%<br>8%<br>9%	Del Papa et al, Bone Marrow Transplant. 2017<br>Van Laar et al JAMA. 2014<br>Farge et al. Br J Haematol. 2002<br>Sullivan et al. NEJM 2018
	Fatigue		18%	Sullivan et al. NEJM 2018
	Concentration problems		-	-
	Depression		29%	Sullivan et al. NEJM 2018

	Infertility (long term)		Fertility impairment: 69% males, 83% females. (allogenic SCT data and depending on age and regimen)[12]	Borgmann-Staudt et al. Bone Marrow Transplant 2012
	Malignancy (long term)		PTLD: 3%<br>Any malignancy: 6%<br>MDS: 6%	Van Laar et al JAMA. 2014<br>Sullivan et al. NEJM 2018
	Other autoimmune disease		Any immune system disorders: 6%	Sullivan et al. NEJM 2018
	Relapse (long term)	Relapse/progression after treatment or the need for additional immunsuppressive treatment due to disease progression or relapse	22% needed DMARD (24m)<br>9% needed DMARD (54m)<br><br>43% during 10 yrs<br><br>24% during first 5 yrs<br>Skin progression in 5%<br>Lung progression in 12%<br><br>25% during upto 26m	Van Laar et al. JAMA 2014<br>Sullivan et al. NEJM 2018<br><br>Nakamura et al. Mod Rheumatol. 2018<br>Bijnen et al. Ann Rheum Dis. 2020<br><br><br><br>Farge et al. Br J Haematol. 2002
MMF				
**Benefits**	Progression/ Event free survival	Improved survival, overall and without occurrence of severe events (for controlled trials: compared to control group)	OS 94% at 24m (compared to 84% without medication[13]<br><br>OS 95% at 5yrs (compared to 86% in patients treated with other medication).[14]	Herrick et al. Ann Rheum. Dis 2016<br><br>Nihtyanova et al. Rheumatology 2007
	Reduced skin thickness (mRSS)	Improvement of skin thickness and extent of skin involvement assessed using the mRSS	72% had skin improvement<br>-5 points on mRSS at 24m[15]<br><br>−4 points on mRSS at 12m	Tashkin et al. Lancet Respir Med. 2016<br><br>Herrick et al. Ann Rheum. Dis 2016
	Improved QoL	Improvement of QoL after treatment assessed using the SF-36 or EQ-5D	Mean SF-36 improved from 66 to 78, particularly the physical component at 12m.[16]	Derk et al. Rheumatology 2009
	Reduced disability	Improvement in daily functioning after treatment, assessed using the HAQ-DI or SHAQ.	HAQ-DI and pain VAS significantly improved at 24m[17]	Vanthuyne et al. Clin Exp Rheumatol. 2007
	Improved interstitial lung disease/lung fibrosis	Improvement of lung function, extent on imaging or symptoms.	Improvement FVC in 72% at 24m[15]<br>Improvement frequent cough from 44% to 41% at 24m[18]<br><br>Improvement of DLco not consistently reported in studies[19]	Tashkin et al. Lancet Respir Med. 2016<br>Tashkin et al. Chest. 2017<br><br><br>Omair et al. PLoS One 2015
	Improved hand function	Improved hand mobility and function, measured with the mHAMIS or subdomain of the SHAQ.	-	-
**Harms**				
	Treatment related mortality	Fatal complications related to the treatment.	4%	Tashkin et al. Lancet Respir Med. 2016
	Gastrointestinal tract disturbance	Side-effects involving any part of the GI tract i.e. diarrhoea, nausea, bloatiness, abdominal pain.	5%<br><br>48%<br>Diarrhoea: 14%<br>Nausea: 9%<br>Abdominal pain: 2%	Nihtyanova et al. Rheumatology 2007<br><br>Omair et al. PLoS One 2015
	Infections		4%<br>26%<br>Pneumonia: 7%	Nihtyanova et al. Rheumatology 2007<br>Omair et al. PLoS One 2015<br>Tashkin et al. Lancet Respir Med. 2016
	Malignancies		2%	Omair et al. PLoS One 2015
	Cytopenia	Including leukopenia, thrombocytopenia, anemia.	7%<br><br>Leukopenia: 6%<br>Thrombocytopenia: 0%<br>Anemia: 12%	Omair et al. PLoS One 2015<br><br>Tashkin et al. Lancet Respir Med. 2016
CYC				
**Benefits**	Progression free survival	Improved survival, overall and without occurrence of severe events (for controlled trials: compared to control group)	EFS 54%, OS 55% at 10yrs<br>EFS 47%, OS 70% at 54m<br><br>–	Van Laar et al. JAMA 2014<br>Sullivan et al. NEJM 2018
	Reduced skin tightness	Improvement of skin thickness and extent of	-8.8 (SD) at 24m<br>-2.2 (median) at 12m	Van Laar et al. JAMA 2014<br>Herrick et al. Ann Rheum. Dis 2016<br>Namas et al. Arthritis Care Res 2018

		skin involvement assessed using the mRSS	-5.4 (SD) at 12m, -7.2 at 24m[20]	
	Increased quality of life	Improvement of QoL after treatment assessed using the SF-36 or EQ-5D	No clinical important difference<br><br>Clinical important difference SF-36 mental domain 33%, physical domain 21% at 12m.[21]	Burt. Lancet. 2011<br><br><br>Khanna Arthritis Rheum. 2007
	Reduced disability	Improvement in daily functioning after treatment, assessed using the HAQ-DI or SHAQ.	31% had clinically significant improvement at 12m	Khanna et al. Arthritis Rheum 2007
	Improved pulmonary function	Improvement of lung function, extent on imaging or symptoms.	+2.5% in FVC at 12m<br>+4.2% in FVC at 12m[22]<br>+2.9% in FVC at 24m<br><br>-2.8% in FVC at 24m (only 12m CYC)<br><br>Frequent cough: 60 to 45% at 24m[18]<br>+7.4% in FVC at 12m	Tashkin et al NEJM 2006<br>Hoyles et al. Arthritis Rheum 2006<br>Tashkin Lancet Respir Med. 2016<br><br>Van Laar et al. JAMA 2014<br><br><br>Tashkin et al. Chest 2017<br>Herrick et al. Ann Rheum. Dis 2016
	Infertility		No impact on fertility of males in transplant patients.[23] No impact of fertility on female patients with lupus.[24]	Perez-Garcia et al. Hum Reprod Update. 2020<br><br>Andreoli et al. Ann Rheum Dis 2017
**Harms**	Treatment related mortality	Fatal complications related to the treatment.	0%<br>0%<br><br>11%	Sullivan et al. NEJM 2018<br>Van Laar et al. JAMA 2014<br><br>Tashkin et al. Lancet Respir Med. 2016
	Cardiac toxicity	Reduced LVEF due to treatment, cardiovascular grade 3 / 4 toxicity	10% at 2yrs<br><br>12% at 26m	Van Laar et al. JAMA 2014<br><br>Sullivan et al. NEJM 2018
	Infection	Infection grade 3 / 4 toxicity	5% of which 1% viral<br>16% at 26m<br>Pneumonia: 5%	Van Laar et al. JAMA 2014<br>Sullivan et al. NEJM 2018<br>Tashkin et al. Lancet Respir Med. 2016
	Relapse (long term)	Relapse/progression after treatment or the need for additional immunosuppressive treatment due to disease progression or relapse	75% at 5 yrs	Sullivan et al. NEJM 2018
	Fertility		Cessation of menses 30%[25]<br><br>Chance of pregnancy dependent on cummulative dose. Time to pregnancy and CYC infusion risk factor for successful pregancy[26]<br><br>Reduced sperm count[27]<br><br>20% erectile dysfunction, 48% abnormal semen analysis[28]	Harward et al. Lupus 2013<br><br>Sen et al. Lupus 2020<br><br><br><br><br>Mouyis et al. Semin ARthritis Rheum 2019<br>Tiseo et al. Int Braz J Urol. 2016
	Malignancy		4% at 2 years<br>0% at 5 years<br><br>0.6% bladder carcinoma at 2yrs[29]	Van Laar et al. JAMA 2014<br>Sullivan et al. NEJM 2018<br><br>Furst et al. Am J Med 2011
	Cytopenia	Decrease of platelets, red blood cells or white blood cells following administration of CYC	Leukopenia: 26% at 12m<br><br>Leukopenia: 41%<br>Thrombocytopenia: 6%<br>Anemia: 18%	Furst et al. Am J Med 2011<br><br>Tashkin et al. Lancet Respir Med. 2016
MTX				
**Benefits**	Progression free survival	Improved survival, overall and without occurrence of severe events (for controlled trials: compared to control group)	OS 94% at 24m (compared to 84% without medication<br><br>OS 84% at 12m, 72% at 36m[30]	Herrick et al. Ann Rheum. Dis 2016<br><br><br>Panopoulos et al. Arthritis Res Ther 2020
	Reduced skin thickness	Improvement of skin thickness and extent of skin involvement assessed using the mRSS	−4 units mRSS at 12m<br><br>- 4 units mRSS at 12m[31]<br>- 4 units mRSS at 12m[32]	Herrick et al. Ann Rheum. Dis 2016<br>Pope et al. Arthritis Rheum. 2001<br>Sumanth et al. Int J Dermatol 2007
	Increased quality of life	Improvement of QoL after treatment assessed using the SF-36 or EQ-5D	-	-

	Reduced disability	Improvement in daily functioning after treatment, assessed using the HAQ-DI or SHAQ.	No significant change in HAQ-DI at 12m. (compared to placebo)	Pope et al. Arthritis Rheum. 2001
	Mouth opening		Improved mouth opening (33-34mm)	Sumanth et al. Int J Dermatol 2007
	Improved pulmonary function	Improvement of lung function, extent on imaging or symptoms.	Significant difference in MTX group at 12m compared to placebo (-4% vs -8%).<br><br>No significant change at 6m	Pope et al. Arthritis Rheum. 2001<br><br>Sumanth et al. Int J Dermatol 2007
	Improved hand function		-	-
	Subjective improvement		Improved binding down: 80%<br>RP: 96%<br>Fingertip ulceration: 89%<br>Hyperpigmentation: 77%<br>Dyspnea: 46% at 6m	Sumanth et al. Int J Dermatol 2007
**Harms**	Treatment related mortality	Fatal complications related to the treatment.	-	-
	Cardiac toxicity	Reduced LVEF due to treatment, cardiovascular grade 3 / 4 toxicity	-	
	Infection	Infection grade 3 / 4 toxicity	Upper respiratory tract: 3%<br>In RA patients: 8% serious infections[33]	Sumanth et al. Int J Dermatol 2007<br>Salliot et al. Ann Rheum Dis. 2009
	Relapse (long term)	Relapse/progression after treatment or the need for additional immunosuppressive treatment due to disease progression or relapse	-	-
	Gastrointestinal tract disturbance	Side-effects involving any part of the GI tract i.e. diarrhoea, nausea, bloatiness, abdominal pain.	Vomiting, nausea: 9%	Sumanth et al. Int J Dermatol 2007
	Alopecia		6% (transient)	Sumanth et al. Int J Dermatol 2007
	Fertility		No effect on fertility in RA patients[34]	De Cock et al. Semin Arthritis Rheum. 2020
	Malignancy		In RA patients: no increased risk	Salliot et al. Ann Rheum Dis. 2009
	Cytopenia	Decrease of platelets, red blood cells or white blood cells following administration of MTX	In RA patients: 5%	Salliot et al. Ann Rheum Dis. 2009
	Liver test disturbance		Increased bilirubin: 3%<br><br>In RA patients: 4% discontinued because of liver toxicity.	Sumanth et al. Int J Dermatol 2007<br>Salliot et al. Ann Rheum Dis. 2009
	Methotrexate pneumonitis	Acute hypersensitivity reaction	In RA patients: 0.4%	Salliot et al. Ann Rheum Dis. 2009

\- no data found in literature

Abbreviations: CMV: cytomegalovirus, CYC: cyclophosphamide, DLco: diffusion capacity of carbon monoxide, DMARD: disease modifying anti-rheumatic drug, EBV: Ebstein Barr virus, EFS: event free survival, EQ-5D: EuroQoL 5 dimensions, FVC: forced vital capacity, GI: gastrointestinal, G-CSF: granulocyte-colony stimulating factor, HAQ-DI: Health Assessment Questionnaire Disability Index, HSV: herper zoster virus, LEVF: left ventricular ejection fraction, MDS: myelodysplastic syndrome, mHAMIS: modified hand mobility in systemic scleroderma, MMF: mycophenolate mofetil, m: months, mRSS: modified Rodnan Skin Score, MTX: methotrexate, OS: overall survival, QoL: Quality of Life, PTLD: post-transplant lymphoproliferative disease, RA: rheumatoid arthritis, RP: Raynaud's phenomenon, RR: risk ratio, SCT: stem cell transplantation, SD: standard deviation, SF-36: short form survey, SHAQ: Scleroderma Health Assessment Questionnaire, VAS: visual analog scale, VZ: varicella zoster, yrs: years

References

1. Burt RK, Shah SJ, Dill K, et al. Autologous non-myeloablative haemopoietic stem-cell transplantation compared with pulse cyclophosphamide once per month for systemic sclerosis (ASSIST): An open-label, randomised phase 2 trial. *Lancet* 2011; 378: 498–506.

2. Van Laar JM, Farge D, Sont JK, et al. Autologous hematopoietic stem cell transplantation vs intravenous pulse cyclophosphamide in diffuse cutaneous systemic sclerosis: A randomized clinical trial. *J Am Med Assoc* 2014; 311: 2490–2498.
3. Sullivan K, Goldmuntz E, Furst D. Autologous Stem-Cell Transplantation for Severe Scleroderma. *N Engl J Med* 2018; 378: 1066–1067.
4. Shouval R, Furie N, Raanani P, et al. Autologous Hematopoietic Stem Cell Transplantation for Systemic Sclerosis: A Systematic Review and Meta-Analysis. *Biol Blood Marrow Transplant* 2018; 24: 937–944.
5. Vonk MC, Broers B, Heijdra YF, et al. Systemic sclerosis and its pulmonary complications in The Netherlands: An epidemiological study. *Ann Rheum Dis* 2009; 68: 961–965.
6. Nakamura H, Odani T, Yasuda S, et al. Autologous haematopoietic stem cell transplantation for Japanese patients with systemic sclerosis: Long-term follow-up on a phase II trial and treatment-related fatal cardiomyopathy. *Mod Rheumatol* 2018; 28: 879–884.
7. Van Bijnen S, De Vries-Bouwstra J, Van Den Ende CH, et al. Predictive factors for treatment-related mortality and major adverse events after autologous haematopoietic stem cell transplantation for systemic sclerosis: Results of a long-term follow-up multicentre study. *Ann Rheum Dis*. 2020. PMID: 32409324
8. Puyade M, Patel A, Lim YJ, et al. Autologous Hematopoietic Stem Cell Transplantation for Behçet's Disease: A Retrospective Survey of Patients Treated in Europe, on Behalf of the Autoimmune Diseases Working Party of the European Society for Blood and Marrow Transplantation. *Front Immunol*; 2021.6; 12: 638709
9. Helbig G, Widuchowska M, Koclęga A, et al. Safety profile of autologous hematopoietic stem cell mobilization and transplantation in patients with systemic sclerosis. *Clin Rheumatol* 2018; 37: 1709–1714.
10. Del Papa N, Onida F, Zaccara E, et al. Autologous hematopoietic stem cell transplantation has better outcomes than conventional therapies in patients with rapidly progressive systemic sclerosis. *Bone Marrow Transplant* 2017; 52: 53–58.
11. Farge D, Marolleau JP, Zohar S, et al. Autologous bone marrow transplantation in the treatment of refractory systemic sclerosis: Early results from a French multicentre phase I-II study. *Br J Haematol* 2002; 119: 726–739.
12. Borgmann-Staudt A, Rendtorff R, Reinmuth S, et al. Fertility after allogeneic haematopoietic stem cell transplantation in childhood and adolescence. *Bone Marrow Transplantation* 2012; 47: 271–276.
13. Herrick AL, Pan X, Peytrignet S, et al. Treatment outcome in early diffuse cutaneous systemic sclerosis: The European Scleroderma Observational Study (ESOS). *Ann Rheum Dis* 2017; 76: 1207–1218.
14. Nihtyanova SI, Brough GM, Black CM, et al. Mycophenolate mofetil in diffuse cutaneous systemic sclerosis - A retrospective analysis. *Rheumatology* 2007; 46: 442–445.
15. Tashkin DP, Roth MD, Clements PJ, et al. Mycophenolate mofetil versus oral cyclophosphamide in scleroderma-related interstitial lung disease (SLS II): a randomised controlled, double-blind, parallel group trial. *Lancet Respir Med* 2016; 4: 708–719.
16. Derk CT, Grace E, Shenin M, et al. A prospective open-label study of mycophenolate mofetil for the treatment of diffuse systemic sclerosis. *Rheumatology* 2009; 48: 1595–1599.
17. Vanthuyne M, Blockmans D, Westhovens R, et al. A pilot study of mycophenolate mofetil combined to intravenous methylprednisolone pulses and oral low-dose glucocorticoids in severe early systemic sclerosis. *Clin Exp Rheumatol* 2007; 25: 287–292.
18. Tashkin DP, Volkmann ER, Tseng CH, et al. Improved Cough and Cough-Specific Quality of Life in Patients Treated for Scleroderma-Related Interstitial Lung Disease: Results of Scleroderma Lung Study II. *Chest* 2017; 151: 813–820.
19. Omair MA, Alahmadi A, Johnson SR. Safety and effectiveness of mycophenolate in systemic sclerosis. A systematic review. *PLoS One*; 2015 1;10(5):e0124205.
20. Namas R, Tashkin DP, Furst DE, et al. Efficacy of Mycophenolate Mofetil and Oral Cyclophosphamide on Skin Thickness: Post Hoc Analyses From Two Randomized Placebo-Controlled Trials. *Arthritis Care Res* 2018; 70: 439–444.
21. Khanna D, Yan X, Tashkin DP, et al. Impact of oral cyclophosphamide on health-related quality of life in patients with active scleroderma lung disease: Results from the scleroderma lung study. *Arthritis Rheum* 2007; 56: 1676–1684.
22. Hoyles RK, Ellis RW, Wellsbury J, et al. A multicenter, prospective, randomized, double-blind, placebo-controlled trial of corticosteroids and intravenous cyclophosphamide followed by oral azathioprine for the treatment of pulmonary fibrosis in scleroderma. *Arthritis Rheum* 2006; 54: 3962–3970.
23. Perez-Garcia LF, Dolhain RJEM, Vorstenbosch S, et al. The effect of paternal exposure to immunosuppressive drugs on sexual function, reproductive hormones, fertility, pregnancy and offspring outcomes: A systematic review. *Hum Reprod Update* 2020; 26: 961–1001.

24. Andreoli L, Bertsias GK, Agmon-Levin N, et al. EULAR recommendations for women's health and the management of family planning, assisted reproduction, pregnancy and menopause in patients with systemic lupus erythematosus and/or antiphospholipid syndrome. *Ann Rheum Dis* 2017; 76: 476–485.
25. Harward LE, Mitchell K, Pieper C, et al. The impact of cyclophosphamide on menstruation and pregnancy in women with rheumatologic disease. *Lupus* 2013; 22: 81–86.
26. Sen M, Kurl A, Khosroshahi A. Pregnancy in patients with systemic lupus erythematosus after cyclophosphamide therapy. *Lupus* 2021; 9612033211021164.
27. Mouyis M, Flint JD, Giles IP. Safety of anti-rheumatic drugs in men trying to conceive: A systematic review and analysis of published evidence. *Semin Arthritis Rheum* 2019; 48: 911–920.
28. Tiseo BC, Cocuzza M, Bonfá E, et al. Male fertility potential alteration in rheumatic diseases: A systematic review. *International Braz J Urol* 2016; 42: 11–21.
29. Furst DE, Tseng CH, Clements PJ, et al. Adverse events during the scleroderma lung study. *Am J Med* 2011; 124: 459–467.
30. Panopoulos S, Chatzidionysiou K, Tektonidou MG, et al. Treatment modalities and drug survival in a systemic sclerosis real-life patient cohort. *Arthritis Res Ther*; 2020 23;22(1):56.
31. Pope JE, Bellamy N, Seibold JR, et al. A randomized, controlled trial of methotrexate versus placebo in early diffuse scleroderma. *Arthritis Rheum* 2001; 44: 1351–1358.
32. Sumanth MK, Sharma VK, Khaitan BK, et al. Evaluation of oral methotrexate in the treatment of systemic sclerosis. *Int J Dermatol* 2007; 46: 218–223.
33. Salliot C, Van Der Heijde D. Long-term safety of methotrexate monotherapy in patients with rheumatoid arthritis: A systematic literature research. *Ann Rheum Dis* 2009; 68: 1100–1104.
34. De Cock D, Brants L, Soenen I, et al. A systematic review on the effect of DMARDs on fertility in rheumatoid arthritis. *Sem Arthritis Rheumat* 2020; 50: 873–878.

Supplementary appendix 2

List of items for Questionnaire Delphi rounds/ Consensus on benefits and harms that should be discussed during the consultation reached in round 3

**Benefits**	**Consensus**	**Harms/cons**	**Consensus**
Progression free survival	**95%**	Treatment related mortality	**79%**
Improvement of quality of life	**77%**	Infections	67%
Improved daily functioning	**77%**	Flare or relapse of disease	63%
Improved pulmonary function	65%	Temporary increase in fatigue	40%
Improved skin thickening	58%	Temporary negative impact on social life	33%
Improved fatigue	42%	Nausea	30%
Improved mobility	28%	Cardiac damage	26%
Improved handfunction	16%	Higher risk to develop malignancy	23%
Improved mood	14%	Less ability to focus	19%
Improved cardiac function	7%	Diarrhea	19%
Improved esophegal function	7%	Erectile dysfunction	14%
		Depression	14%
		Infertility	12%
		Hair loss	12%
		Hemorrhagic cystitis	12%
		Other autoimmune disease	9%
		Increased disease activity (G-CSF)	7%
		Neuropathic pain	5%
		Cytokine storm	5%
		Hormonal imbalance	5%
		Mouth ulcers	5%
		Temporary decreased mobility	2%
